# Perceived stress across the midlife: longitudinal changes among a diverse sample of women, the Study of Women’s health Across the Nation (SWAN)

**DOI:** 10.1186/s40695-018-0032-3

**Published:** 2018-03-16

**Authors:** Elizabeth Hedgeman, Rebecca E. Hasson, Carrie A. Karvonen-Gutierrez, William H. Herman, Siobán D. Harlow

**Affiliations:** 1 Department of Epidemiology, School of Public Health, University of Michigan, 6610B SPH I, 1415 Washington Heights, Ann Arbor, MI 48109-2029 USA; 2 School of Kinesiology, School of Public Health, University of Michigan, Ann Arbor, USA; 3 Department of Internal Medicine, School of Public Health, University of Michigan, Ann Arbor, USA

**Keywords:** Women’s health, Stress, Minority health/disparities/SES, Aging, Menopausal transition, Epidemiology

## Abstract

**Background:**

In women, midlife is a period of social and physiological change. Ostensibly stressful, cross-sectional studies suggest women experience decreasing stress perceptions and increasing positive outlook during this life stage. The aim of this paper was to describe the longitudinal changes in perceived stress as women transitioned through the midlife.

**Methods:**

Premenopausal women (*n* = 3044) ages 42–52 years at baseline, were recruited from seven sites in the Study of Women’s Health Across the Nation, and followed approximately annually over 13 visits with assessment of perceived stress and change in menopausal status. Longitudinal regression models were used to assess the effects of age, menopausal status and baseline sociodemographic variables on the trajectory of perceived stress over time.

**Results:**

At baseline, mean age was 46.4 ± 2.7 years; participants were white (47%), black (29%), Hispanic (7%), Japanese (9%), or Chinese (8%). Hispanic women, women with lesser educational attainment, and women reporting financial hardship were each more likely to report high perceived stress levels at baseline (all *p* < 0.0001). After adjustment for baseline sociodemographic factors, perceived stress decreased over time for most women (*p* < 0.0001), but increased for both Hispanic and white participants at the New Jersey site (*p* < 0.0001). Changing menopausal status was not a significant predictor of perceived stress.

**Conclusions:**

Self-reported stress decreased for most women as they transitioned across the midlife; changing menopausal status did not play a significant role after adjustment for age and sociodemographic factors. Future studies should explore the stress experience for women by racial / ethnic identity and demographics.

**Electronic supplementary material:**

The online version of this article (10.1186/s40695-018-0032-3) contains supplementary material, which is available to authorized users.

## Background

The midlife, bounded by young adulthood and old age, has heretofore received only limited scientific attention. Modern social scientists place the beginning of midlife at 35 or 40 years of age, to highlight the period when most adults have finished schooling, entered the workforce, and embarked into marriage with childbearing and rearing [1] – a period of “life past the initial putting together [2].” Clinically this life phase coincides with the age at which chronic conditions begin to appear, an age that can vary by cultural and sociodemographic identity [3]. When asked themselves, adults cite midlife as beginning anywhere from 35 to 45 and ending around 55–60 years of age [2, 4, 5].

For modern women 40–65 years of age, these middle years are marked by the potential for profound social and physiological changes [6]. Households are changing, with children leaving and “boomerang” children returning [7, 8]. Aging parents may require more care as their health and functioning decline. Workplace stress may increase with the attainment of seniority, additional job strain, and concomitantly increasing time demands [9, 10]. The menopausal transition – a period beginning in the early forties, marking reproductive senescence, changing estrogen levels, and ultimate cessation of the menstrual cycle – can bring vasomotor and genitourinary symptoms, disrupted sleep cycles and mood changes [2, 11–14]. Though the ‘midlife crisis’ has been largely debunked [15], the midlife years appear to be a period ripe for stress. Previous work has demonstrated that positive affect – a measure of positive mood and outlook – was significantly lower in midlife women (ages 35–64 years in 1995–1996) as compared to younger and older women, with relationship stress and occupational stress found to be strong drivers of the observed dissatisfaction [16, 17].

And yet, perhaps contrary to expectation, research suggests that perceived stress – a self-reported, subjective measure of individual control and coping – decreases, and quality of life increases, through midlife in some populations. Among nearly 14,000 women ages 40–55 years, contacted in 1994 for the Study of Women’s Health Across the Nation (SWAN) cross-sectional screening study, increased age was positively associated with quality of life for white and black women, though not for Chinese, Hispanic or Japanese women [18]. Similar cross-sectional results from the first wave of the Midlife Development in the United States (MIDUS; 1995–1996) study suggest that overall quality of life reaches a nadir in the late 30s to early 40s, only to increase through the remaining midlife and beyond [19]. Cross-sectional studies from both the United States (1983) and United Kingdom (circa 2006) suggest that levels of perceived stress decrease over the entire lifespan for all race / ethnicities [20, 21]. Corresponding with these cross-sectional findings of lower stress perception with age, the longitudinal Melbourne Women’s Midlife Health Project of Australian-born midlife women (ages 45–55 in 1991) found that negative moods – feelings of tension, confusion, helplessness, loneliness, insignificance – decreased significantly over the 11 years of follow-up [22]. However, missing from this literature is a longitudinal assessment of perceived stress, particularly across the midlife.

The aim of this study was to describe the longitudinal reports of perceived stress as women transitioned through the midlife in the SWAN cohort. Specific hypotheses, based on the findings from prior research, were that perceived stress (i) would decrease over time for some, but not all women, due to differing racial / ethnic experiences of aging, and (ii) would increase as women progressed through perimenopause, but generally decrease with age. Socioeconomic factors were included in models as modifying factors expected to influence perceived stress. Secondary data were obtained from this large, sociodemographically diverse cohort of women, with individual perceived stress assessed at multiple points over 15 years and 13 visits. Potential differences in the experience of perceived stress by race / ethnicity, adjusted for socioeconomic status, and whether stress profiles were influenced by stage of the menopausal transition, considered a key biological hallmark of this lifestage, were assessed for longitudinal differences over time.

## Methods

### Study population

A full description of the Study of Women’s Health Across the Nation (SWAN) longitudinal cohort and methodology has been published in detail elsewhere [23]. Briefly, SWAN was instituted in 1996 as an observational cohort study of women, their lifestyles, and their health through the menopausal transition with longitudinal follow-up to determine outcomes over time. Eligibility was based on age (42–52 years), self-reported race / ethnicity, and reproductive status (not pregnant or lactating; at least one menstrual cycle in previous three months; uterus and at least one ovary intact; not taking exogenous hormones affecting ovarian function at time of enrollment). Study sites – located in Boston, Massachusetts (MA); Chicago, Illinois (IL); Southeast Michigan (MI); Los Angeles, California (CA); Newark, New Jersey (NJ); Pittsburgh, Pennsylvania (PA); and Oakland, CA – invited recruitment from white, black, Hispanic, Chinese and Japanese communities. All sites recruited white participants, four sites recruited black participants (MA, MI, IL, PA) and one site each recruited Chinese (Oakland, CA), Japanese (Los Angeles, CA) or Hispanic (NJ) participants. At baseline, the full study included 3302 women. Women were followed approximately annually for 13 visits with study participation at 74.5% by visit 13.

For this analysis, women were excluded if they had fewer than two perceived stress scores (*n* = 253) or experienced a pregnancy (*n* = 5) over follow-up. The final analytical sample included 3044 women. Data from the NJ site were truncated at visit five due to an interruption in site operations, affecting 108 white and all 212 Hispanic women.

### Variables

Age, self-reported race / ethnicity, educational attainment (less than high school, high school degree [or equivalent], college degree, post-college training) and smoking status (current smoker yes or no) were ascertained by questionnaire at baseline for all participants. Baseline financial hardship was estimated by self-report to the question: “How hard is it for you to pay for the very basics like food, housing, medical care, and heating”. Available responses were ‘Very Hard’, ‘Somewhat Hard’ and ‘Not very hard at all’. Baseline physical measures including height (centimeters), weight (kilograms) and lightly-clothed waist circumference (in centimeters) were assessed by trained staff during the clinic visit. Body mass index (BMI) was calculated as weight (kg) divided by height (cm) squared.

Perceived stress was self-reported at each visit using the four-item Perceived Stress Scale questionnaire (PSS4) developed and validated by Cohen et al. [20, 24]. PSS4 questions included:

1. In the past two weeks, how often have you felt you were unable to control the important things in your life?
2. In the past two weeks, how often have you felt confident about your ability to handle your personal problems?
3. In the past two weeks, how often have you felt that things were going your way?
4. In the past two weeks, how often have you felt difficulties were piling up so high that you could not overcome them?

Participants indicated the frequency they experienced each of the four stressful situations using a 5-point Likert scale (1 = never, 2 = almost never, 3 = sometimes, 4 = fairly often, 5 = very often). For scoring total perceived stress, responses to negative questions were summed with the reverse of the responses to positive questions, yielding a composite score ranging from 4 to 20. Larger PSS4 scores indicated increased time experiencing stressful situations in the prior two weeks. Perceived stress questions were asked at baseline (year 0) and each follow-up visit, for a total of 13 possible measurements. The mean number of available perceived stress scores per woman was 10.2 (median: 12, range: 2–13); 15.6% had five or fewer perceived stress scores.

Menopausal status was assessed at each visit based on participant’s report of menstrual irregularity [25] or complete cessation of cycles, plus self-reported information on hysterectomy and / or oophorectomy and current hormone use. Menopausal status was coded as premenopausal (menses has occurred in previous 3 months with no change in predictability over past 12 months), early perimenopausal (menses has occurred in previous 3 months, but with less predictability), late perimenopausal (menses has occurred in previous 12 months, but without menses in previous 3 months) or postmenopausal (no menses in past 12 months and / or both ovaries removed). Unknown menopausal status due to hormone use or hysterectomy was collapsed into a single ‘unknown’ category.

### Statistical analysis

Baseline descriptive information was compared for all participants and by baseline reported perceived stress level (categorized as low [≤ 25th percentile], moderate, high [≥ 75 percentile]). Women without a baseline PSS score (*n* = 86) were not included in analyses focused on stress at baseline, but were included in the longitudinal models of perceived stress. Logistic regression, adjusting for age, was used to assess the association of sociodemographic variables with high (versus low + moderate) perceived stress at baseline. To assess for potential bias due to selective loss of participants reporting higher baseline perceived stress, linear regression, adjusting for age, was used to test the difference in baseline perceived stress by loss to follow-up status over the 13 visits.

To guide modeling, change in mean perceived stress was first explored graphically by age, stratified by selected sociodemographic variables expected to contribute to perceived stress (race / ethnicity, educational attainment, baseline financial hardship, site of recruitment). For graphing crude means, age was truncated at 65 years (55 years for Hispanic women) to prevent leverage in slope estimation due to cohort attrition and the smaller numbers of women at the upper tail of the age distribution.

A linear mixed model was examined to understand the contribution of sociodemographic variables and menopausal status to change in perceived stress over time. Variables of interest were first reviewed individually for their effects on perceived stress. Model building was performed sequentially, using a forward stepwise approach, with statistical significance of added variables assessed by variable significance and model fit tested by Likelihood Ratio with alpha set to 0.05. Appropriateness of random effects in models were tested using restricted maximum likelihood and mixed effects were tested using maximum likelihood. An unstructured variance-covariate matrix was assumed. All models incorporated race / ethnicity and age, centered at 42 years, as a time-varying variable and included a random slope for age. Potential interactions of longitudinal age with sociodemographic variables were evaluated to assess differences in slope. Additional interactions with race / ethnicity and socio-economic variables were assessed in separate models, but small cell sizes resulted in model instability.

Final models were assessed for appropriate specification by review of the errors from the random effects (age) as well as the conditional errors for the fixed effects. All errors were assessed for normality graphically. All graphing and statistics were performed using SAS version 9.4 (SAS Institute, Cary, NC).

## Results

### Baseline characteristics

At baseline, the 3044 women eligible for this analysis were a mean age of 46.4 years (range: 42.0–53.0 years) with a racial / ethnic distribution of 47.4% white, 28.7% black, 8.9% Japanese, 8.0% Chinese and 7.0% Hispanic (Table 1). The majority of the cohort (> 90%) had obtained at least a high school degree while 44.1% had attained a college degree or higher. Financial difficulty was reported by nearly 40% of women, with 8.7% reporting that it was ‘very hard’ to pay for the basics of living. Among the 2958 women reporting perceived stress at baseline, mean perceived stress score was 8.5 (median: 8.0, range: 4–19). Characteristics of 86 women without a baseline perceived stress score are available in Additional file 1: Table S1.

**Table 1:** Population characteristics by baseline perceived stress score

			Category of Baseline Perceived Stress^ab^		
	N	Overall (*n* = 3044)	Low (*n* = 844)	Moderate (*n* = 1352)	High (*n* = 762)
Perceived Stress^c^	2958	8.5 ± 2.9	5.1 ± 0.8	8.5 ± 1.1	12.4 ± 1.5
Age^c^	3044	46.4 ± 2.7	46.3 ± 2.7	46.4 ± 2.7	46.2 ± 2.7
Race / Ethnicity (%)					
White	1443	–	29.1	48.1	22.8
Black	874	–	32.3	39.0	28.7
Hispanic	212	–	18.0	31.0	51.0
Japanese	272	–	22.7	54.6	22.7
Chinese	243	–	26.3	59.4	14.3
Education (%)					
Less than High School	191	–	15.4	35.1	49.5
High School	1496	–	26.6	44.0	29.3
College Degree	627	–	30.0	48.5	21.5
Post-College	705	–	34.5	49.9	15.6
Difficulty paying for Basics (%)					
Very Hard	263	–	10.0	38.6	51.4
Somewhat Hard	899	–	17.4	44.2	38.5
Not hard	1865	–	36.5	47.3	16.2
Site of Recruitment (%)					
PA	439	–	29.9	42.8	27.3
MI	503	–	30.7	40.0	29.3
MA	424	–	25.1	51.6	23.4
IL	433	–	34.4	41.8	23.8
Oakland, CA	442	–	27.1	54.9	18.1
Los Angeles, CA	483	–	30.4	50.7	18.9
NJ	320	–	18.7	36.0	45.3
Smoking Status (%)					
Non-Smoker	2522	–	29.0	47.6	23.4
Smoker	498	–	26.6	36.5	36.9
BMI (kg/m^2^)^c^	3009	28.2 ± 7.2	27.9 ± 6.8	27.9 ± 7.1	29.3 ± 7.8
Waist Circumference (cm)^c^	3012	86.1 ± 16.1	85.4 ± 15.6	85.6 ± 15.7	88.7 ± 17.2

^a^Categorical variable *rows* sum to 100%. Numbers may not sum to 100 due to rounding

^b^Note that 86 women had missing baseline PSS4 scores

^c^Mean ± SD

At baseline, Hispanic women were significantly more likely to report high perceived stress as compared to any other race / ethnicity (all comparisons *p* < 0.0001), while Chinese women were significantly less likely to report high stress (*p* < 0.0001 for white, black and Hispanic women, *p* = 0.0185 for Japanese women) (Table 1). Women reporting higher levels of financial hardship were more likely to report high perceived stress than women reporting some or no financial hardship (*p* = 0.0003 and < 0.0001, respectively); and women without a high school diploma were significantly more likely to report high perceived stress than women with a high school diploma, college or other advanced degree (all *p* < 0.0001). Likewise, women who were current smokers were more likely to report high levels of perceived stress as compared to women who were not (*p* < 0.0001), and women with increased BMI or waist circumference were also more likely to report high perceived stress (*p* < 0.0001 for each).

### Perceived stress and increasing age

**Fig. 1:**
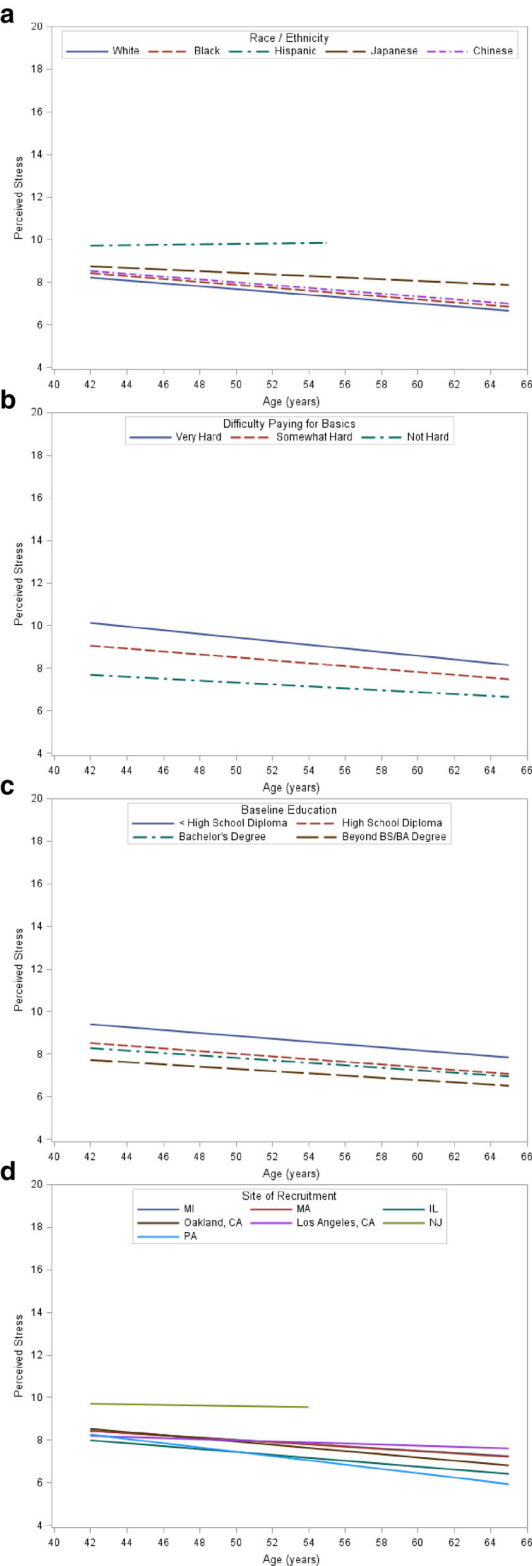
Change in perceived stress over age; age is truncated at 65 years (55 years for Hispanic women) to prevent leverage due to cohort attrition and small numbers. Note the data truncation due to New Jersey site limitation. **a** Race / ethnicity includes all eligible women. **b** Baseline difficulty paying for the basics; New Jersey participants omitted. **c** Baseline education; New Jersey participants omitted. **d** Site of recruitment includes all eligible women

Mean cohort age increased to 62.0 years at the 13th follow-up visit while unadjusted mean perceived stress scores declined by − 0.06 ± 0.00 points with each increased year of age. No difference was seen in baseline perceived stress between women retained and those who died or were lost to follow-up (8.4 ± 2.9 vs 8.5 ± 3.0, respectively, *p* = 0.38). Trajectories for change in perceived stress with age are displayed in Fig. 1a-d, by race / ethnicity, educational attainment, financial hardship, and site of recruitment. Corresponding with the baseline results, women with less educational attainment, women reporting increased financial hardship and women recruited from NJ had higher mean reported levels of perceived stress than their counterparts. In addition, mean perceived stress was observed to decline with age across all sociodemographic categories with the exception of Hispanic women.

Unadjusted regressions for each variable and the final multivariable regression model evaluating the effects of age, menopausal status, race / ethnicity, educational attainment, baseline financial hardship and site of recruitment on longitudinal change in perceived stress are displayed in Table 2. In the final multivariate regression model, women reporting financial hardship and with lesser attained education reported significantly higher levels of perceived stress at baseline as compared to women reporting no financial hardship or training beyond a college degree. Only Japanese race / ethnicity remained as a statistically significant predictor of higher perceived stress after adjustment for financial hardship and educational attainment. Interactions between financial strain and age suggested that moderate and severe baseline financial hardship were associated with a steeper decline in perceived stress over time as compared to no financial hardship. Though mean reported perceived stress decreased over time for most women, for white and Hispanic women located in NJ, perceived stress increased (0.07 ± 0.03 points with each increased year of age) over the five available visits for this site. For interpretation purposes, within this cohort a 42-year-old white woman living near Pittsburgh, with a high school diploma and no reported baseline financial hardship (the ‘reference category’) had a perceived stress score of 7.93, that decreased by 0.10 points over each increasing year of age. In comparison, a Japanese woman of the same age, living near Los Angeles, with a high school education and no baseline financial hardship, reported a perceived stress of 8.17 that decreased by 0.01 points each year, and a Hispanic woman of the same age, living near New Jersey, without a high school education and no baseline financial hardship had a mean perceived stress score of 8.05 that increased by 0.11 points each year.

**Table 2:** Unadjusted and fully adjusted random effects model explaining perceived stress over increasing age

	Unadjusted Parameters		Fully Adjusted Model*	
	β (95% CI)	P (Type 3)	β (95% CI)	P (Type 3)
Intercept	–	–	7.93 (7.65, 8.20)	–
Age	−0.06 (− 0.07, − 0.06)	< 0.0001	−0.10 (− 0.12, − 0.08)	< 0.0001
Race / Ethnicity		< 0.0001		< 0.0001
White	REF		REF	
Black	0.16 (−0.02, 0.35)		−0.06 (− 0.26, 0.14)	
Hispanic	2.06 (1.73, 2.39)		−0.33 (− 0.86, 0.20)	
Japanese	0.71 (0.43, 0.99)		0.84 (0.47, 1.21)	
Chinese	0.31 (0.01, 0.61)		0.28 (−0.11, 0.67)	
Difficulty paying for Basics (%)		< 0.0001		< 0.0001
Not hard	REF		REF	
Somewhat Hard	1.26 (1.09, 1.43)		1.28 (1.06, 1.51)	
Very Hard	2.20 (1.92, 2.48)		2.37 (2.00, 2.74)	
Education		< 0.0001		0.0016
Less than High School	1.37 (1.03, 1.71)		0.34 (−0.02, 0.69)	
High School	REF		REF	
College Degree	−0.23 (− 0.43, − 0.02)		−0.02 (− 0.22, 0.18)	
Post-College Degree	−0.79 (− 0.99, − 0.60)		−0.32 (− 0.52, − 0.12)	
Site of Recruitment		< 0.0001		0.0487
PA	REF		REF	
MI	0.67 (0.40, 0.95)		−0.20 (−0.54, 0.15)	
MA	0.59 (0.3, 0.87)		0.01 (−0.34, 0.37)	
IL	0.02 (−0.27, 0.31)		−0.04 (− 0.40, 0.32)	
Oakland, CA	0.44 (0.15, 0.72)		0.03 (−0.39, 0.44)	
Los Angeles, CA	0.61 (0.33, 0.89)		−0.59 (− 0.99, − 0.18)	
NJ	2.21 (1.89, 2.53)		0.11 (−0.47, 0.70)	
Menopausal Status		< 0.0001	–	–
Pre	REF		–	–
Early peri	−0.20 (−0.29, − 0.10)		–	–
Late peri	−0.30 (− 0.42, − 0.17)		–	–
Post	− 0.67 (− 0.76, − 0.58)		–	–
Unknown	−0.33 (− 0.47, − 0.20)		–	–
Age * Difficulty paying for Basics Interaction	–	–		< 0.0001
Age*Not hard	–	–	REF	
Age*Somewhat Hard	–	–	−0.02 (− 0.04, − 0.01)	
Age*Very Hard	–	–	− 0.05 (− 0.08, − 0.02)	
Age * Site Interaction	–	–		
Age*PA	–	–	REF	< 0.0001
Age*MI	–	–	0.08 (0.05, 0.10)	
Age*MA	–	–	0.06 (0.03, 0.08)	
Age*IL	–	–	0.02 (− 0.00, 0.05)	
Age*Oakland, CA	–	–	0.04 (0.02, 0.07)	
Age*Los Angeles, CA	–	–	0.09 (0.06, 0.11)	
Age*NJ	–	–	0.21 (0.16, 0.26)	

*The multivariate model includes all variables listed; menopausal status was not statistically significant (*p* > 0.05) in the final model

When menopausal status was added to the final adjusted model with longitudinal age, model fit increased significantly (Likelihood Ratio *p* < 0.00001). Results suggested that progression through each stage of the menopausal transition (from pre-menopause onward) was associated with a further decrease in perceived stress, however the menopausal status variable did not reach statistical significance (*p* = 0.5203; data not shown) and thus was omitted from the final model.

## Discussion

This study is one of the first to describe longitudinal change in perceived stress levels in a multi-ethnic sample of midlife women in the United States. Mean levels of self-reported stress, as measured annually by Cohen’s Perceived Stress Scale, decreased for most women as they transitioned across the midlife. Compared to similar black, white and Chinese women within SWAN, mean levels of perceived stress decreased in a more attenuated fashion for Japanese women, but increased over time for white and Hispanic women living in New Jersey. In addition, women with lower educational attainment, and in particular, baseline financial hardship, consistently reported higher levels of perceived stress, though this difference diminished with time. After adjustment for other sociodemographic variables, race / ethnicity was a significant predictor of increased perceived stress for only Japanese women. Changing menopausal status did not play a significant role in change in perceived stress after adjustment for age and sociodemographic factors.

Cross-sectional studies performed both in the United States and the United Kingdom have suggested that perceived stress decreases with age. A 1983 population-based survey of adults in the United States reported a mean PSS4 of 4.9 ± 3.0 for adults ages 18–29 years, 4.4 ± 2.9 for adults ages 45–54 years and 4.0 ± 3.0 for adults ages 65 years and older using a 0–15 scale (corresponding to mean PSS4 scores of 8.9, 8.4 and 8.0, respectively, on the 4–20 scale used here) [20]. Reported perceived stress was higher among women compared to men, Hispanics and blacks as compared to whites, and increased with lower annual income and educational attainment. Similarly, a more recent cross-sectional review of reported perceived stress from individuals ages 16–85 years living in the United Kingdom indicated that younger age, female sex, reduced social support and black, Asian (Indian, Pakistani, Bangladeshi, other) or mixed (as compared to white) race were associated with higher PSS4 scores [21].

While the unadjusted results indicated that women of non-white ethnicity or with lower socioeconomic means tended to report higher perceived stress, supporting the findings from the above referenced studies, the *adjusted* analyses presented here indicated that black women and Hispanic women reported lower perceived stress at baseline as compared to similar white, Japanese and Chinese women in SWAN. Although these differences did not reach statistical significance, our findings are in contrast to studies of SWAN participants that indicate that black women (in particular) and Chinese women report higher levels of perceived discrimination and unfair treatment than their peers, and that these reports are tied to increased biological stress reactivity and decreased mental and physical health [26–28]. This paradox – lower perceived stress reports among subgroups showing higher biological response to stressors – may be explained by a tendency for women with lower social standing to internalize and normalize stressors that are experienced frequently [29–31]. For example, Lee and Bierman found that, in older adults experiencing discrimination, decreased social status was associated with fewer outward expressions of anger, but more suppressed or internalized experiences of anger; the authors theorized that anger suppression was a coping mechanism and a method to de-escalate potentially dangerous situations [30]. These finding with the SWAN cohort are intriguing and warrant further investigation.

In further comparison to the cross-sectional studies, the work presented here indicates that there are variations in the rate of change of perceived stress in some subgroups of women and, moreover, that not all individuals experience decreases over time. The faster rate of decrease in perceived stress scores for women initially in the higher categories of baseline financial hardship may be due to alleviation of the stressor as women age into retirement [32] or may reflect acute baseline financial stressors associated with only temporary increases in perceived stress. Conversely, the results may reflect selective cohort loss over time among women reporting higher financial hardship, although mean baseline perceived stress scores did not vary by attrition status. Curiously, while our results indicate that perceived stress decreased for all women to some varying degree as they aged across the midlife, Hispanic and white women living in or near Newark, NJ reported increasing perceived stress over the course of their five visits from baseline. Due to the interruption of activities at the NJ site, it is impossible to determine whether the observed perceived stress trajectory would have continued to increase or reverse course over the remaining 8 visits. Notably, the fifth follow-up occurred primarily in 2001/2002, and results may have been influenced by the World Trade Center bombing in September 2001 [33]. Moreover, as Hispanic women were recruited only from this site, it is impossible to disentangle the site effect from the experience of being a midlife Hispanic woman in the United States.

Our results found no increase in perceived stress associated with changing menopausal status after adjustment for aging and sociodemographic characteristics. These findings are in contrast to existing cross-sectional work and some longitudinal work suggesting that the menopausal transition is associated with higher stress and depression. Freeman et al. found that higher perceived stress was independently associated with higher menopausal symptom severity including: hot flushes, poor sleep quality, depression and general aches and stiffness [34]. Though these findings are intriguing, they excluded assessment of general socioeconomic status – obscuring the role of general life stressors during the experience of menopause [35]. More recently, when adjusting for study visit, Falconi et al. found that early and late perimenopause were significantly associated with increases in perceived stress [36], but they did not adjust for age or sociodemographic indicators. Prior publications have indicated that women who proceed through menopause at an earlier age are socioeconomically disadvantaged [37–39] and already prone to increased life stress [40]. Woods et al., in longitudinal analyses from the Seattle Midlife Women’s Study, which included predominately white women but adjusted for age, found that factors such as employment and health status, but also pre-existing mood disturbances, were the only significant predictors of perceived stress over a decade, and not the menopausal transition itself [41]. Our findings are consistent with Woods et al. as we found that the role of socioeconomic factors such as educational attainment, employment and financial hardship were stronger predictors of perceived stress over midlife than the menopausal transition itself in this larger, more diverse sample of midlife women. These findings may suggest that women experience the menopausal transition as a series of acute stressors (e.g., hot flashes, sleep disturbances) that can be attenuated by chronic, socioeconomic-based life stressors, however further work would be necessary to substantiate this theory.

Explanations for the observed decreases in perceived stress with age are suggestive yet incomplete. Research suggests that older adults show more maturity and regulation of emotion [42, 43], leading to increased feelings of optimism and fewer symptoms of psychological distress than younger adults [44, 45], however the cross-sectional nature of most extant studies can not rule out a cohort effect based on era of birth. Beyond changes in the appraisal and regulation of stress, changing life roles with age, such as retirement or the relinquishment of parenting, may lead to the occurrence of fewer stressful events even as individual health may be declining [46]. Focus groups performed with women in the United States suggest that the midlife is a time of reduced child-rearing responsibilities leading to role restructuring, more control over one’s time, and an increased sense of personal power and freedom [47–50] – concepts embedded in Cohen’s Perceived Stress Scale. Finally, recent longitudinal work performed by Lachman et al. for the Midlife in the United States (MIDUS) study shows that life satisfaction significantly increases across the midlife decades (4th to 5th, 5th to 6th decades) [5], again corresponding with the decreases in perceived stress seen in this work.

Despite the decreasing perception of stress with age, individuals who report relatively greater stress at the start of the midlife continue to report higher stress levels as they age, an important finding given that more highly stressed individuals are at greater health risk than their less-stressed peers. Arnold et al. found that high or moderate baseline perceived stress increased mortality risk for adults hospitalized with acute myocardial infarction (high/moderate vs. low PSS4 score: aHR = 1.42 [95% CI = 1.15–1.76]) [51]. Investigators for the REGARDS study found that, for individuals with household incomes < $35,000/ yr., baseline PSS4 score was associated with all-cause mortality risk (high vs. no stress: aOR = 1.55 [95%CI: 1.31–1.82]), and marginally associated with incident coronary heart disease (high vs. no stress: aOR = 1.29 [95%CI: 0.99–1.69]) [52]. Similarly, Aggarwal et al. identified increased baseline perceived stress, as measured by a modified ‘PSS6’ score, to be predictive of future cerebral infarct in older adults (high vs. low PSS6 score: aOR = 1.94, 95% CI = 1.11–3.40) [53]. As individuals in lower socioeconomic and sociocultural strata are more at risk of adverse health outcomes such as diabetes, stroke and myocardial infarct [54–59], it is plausible that individual perception and internal assimilation of stress is one of many factors directly influencing health [60]. Future work will assess whether the women of SWAN who report higher perceived stress, and lower socioeconomic means, are more at risk of adverse outcomes.

The primary limitation of this study was our limited ability to understand perceived stress among women reporting the highest levels over time. Hispanic women, women reporting extreme financial hardship at baseline, and those with the least education, each comprised less than 10% of the study sample, limiting power and preventing analyses to disentangle potential interactions among these subgroups. Similarly, the disruption of operations at the NJ site, a site situated to recruit Hispanic women and women of lower socioeconomic means, prevented a complete review of change in reported perceived stress over time at that site. Only baseline financial hardship was assessed in these models as it was not measured at every follow-up visit. Fluctuating hardship levels may explain additional variability over time. It is also worth noting that we are ascribing self-reports of perceived stress over a two-week period to women’s perceptions over the course of a year, ruling out a detailed assessment of stress that women may feel on a day-to-day basis. This broader view of stress, in addition to our exploration of menopausal *stages* versus menopausal *symptoms*, may have precluded the assessment of the impact of stressors that fluctuate on a daily basis, such as from vasomotor symptoms. Finally, we have chosen to review and model mean change over time, which may obscure subtle differences in trajectories of stress that are non-linear; a subject worth further exploration. Nonetheless, the analyses presented, incorporating the diverse cohort from the SWAN longitudinal study, provide important information about stress over the midlife and menopausal transition.

## Conclusion

In conclusion, this study found that the perception of stress decreased over time for the majority of this diverse set of midlife women in the United States. Perceived stress increased for the Hispanic and white women recruited from New Jersey, and was consistently greater among women with lesser education attainment and women experiencing financial difficulty. Concomitant with the increased reporting of stress, those with higher stress were more likely to smoke and have higher BMIs at baseline. While we are limited to observing the change in stress over the thirteen years of study – and solely within women – our results add credence to the original surveys performed by Cohen et al. [20, 24], and provide further evidence that decreases in stress are truly age-related and not related to era of birth. Future work is necessary to further explore the stress experience for women in the United States, especially as it varies by racial / ethnic identity, but also to assess longitudinal trajectories of stress that are non-linear or unchanging over time, change with changing life roles, and to tie the observed perceived stress differences with adverse mental and clinical outcomes.

## Additional file

Additional file 1: **Table S1.** Population characteristics by baseline perceived stress score availability. (DOCX 17 kb)
