# Supplementary material for: Perceived stress across the midlife: longitudinal changes among a diverse sample of women, the Study of Women’s health Across the Nation (SWAN)
Source: Womens Midlife Health. 2018 Mar 16;4:2. doi: 10.1186/s40695-018-0032-3 (PMC6027744; doi:10.1186/s40695-018-0032-3)
Supplement: Supplementary file 1 — Table S1. Population characteristics by baseline perceived stress score availability. (DOCX 17 kb) [file 40695_2018_32_MOESM1_ESM.docx]

Additional file 1: **Table S1** Population characteristics by baseline perceived stress score availability

|  | **Baseline Perceived Stress Score Availability** | | **P*** |
| --- | --- | --- | --- |
|  | **Available**  **(n = 2958)** | **Not**  **(n = 86)** |  |
| Age (mean ± SD) | 46.4 ± 2.7 | 46.7 ± 2.6 | 0.3083 |
|  |  |  |  |
| Race / Ethnicity (%) |  |  | <0.0001 |
| White | 47.8 | 33.7 |  |
| Black | 29.1 | 16.3 |  |
| Hispanic | 6.8 | 14.0 |  |
| Japanese | 8.8 | 14.0 |  |
| Chinese | 7.6 | 22.1 |  |
|  |  |  |  |
| Education (%) |  |  | 0.1152 |
| Less than HS | 6.18 | 12.16 |  |
| High School | 49.6 | 48.7 |  |
| College Degree | 20.7 | 23.0 |  |
| Post-College | 23.5 | 16.2 |  |
|  |  |  |  |
| Difficulty paying for Basics (%) |  |  | 0.0091 |
| Very Hard | 8.4 | 18.2 |  |
| Somewhat Hard | 29.7 | 29.9 |  |
| Not hard | 61.9 | 52.0 |  |
|  |  |  |  |
| Site of Recruitment (%) |  |  | <0.0001 |
| Michigan | 16.7 | 9.3 |  |
| Massachusetts | 13.9 | 15.1 |  |
| Illinois | 14.5 | 5.8 |  |
| Oakland, CA | 14.2 | 24.4 |  |
| Los Angeles, CA | 15.9 | 14.0 |  |
| New Jersey | 10.1 | 23.3 |  |
| Pennsylvania | 14.6 | 8.1 |  |
|  |  |  |  |
| Smoking Status (%) |  |  | 0.7276 |
| Non-Smoker | 83.5 | 84.9 |  |
| Smoker | 16.5 | 15.12 |  |
|  |  |  |  |
| BMI (kg/m^2^, mean ± SD) | 28.3 ± 7.2 | 25.6 ± 6.4 | 0.0008 |
|  |  |  |  |
| Waist Circumference  (cm, mean ± SD) | 86.3 ± 16.1 | 79.5 ± 12.8 | <0.0001 |

*P values comparing women with perceived stress scores at baseline versus those without were calculated using t-tests for continuous and chi square tests for categorical variables.
